# Dual-Energy CT of the Heart: A Review

**DOI:** 10.3390/jimaging8090236

**Published:** 2022-09-01

**Authors:** Serena Dell’Aversana, Raffaele Ascione, Marco De Giorgi, Davide Raffaele De Lucia, Renato Cuocolo, Marco Boccalatte, Gerolamo Sibilio, Giovanni Napolitano, Giuseppe Muscogiuri, Sandro Sironi, Giuseppe Di Costanzo, Enrico Cavaglià, Massimo Imbriaco, Andrea Ponsiglione

**Affiliations:** 1 Department of Radiology, Santa Maria delle Grazie Hospital, ASL Napoli 2 Nord, 80078 Pozzuoli, Italy; 2 Department of Advanced Biomedical Sciences, University of Naples Federico II, 80131 Naples, Italy; 3 Department of Medicine, Surgery and Dentistry, University of Salerno, 84081 Baronissi, Italy; 4 Coronary Care Unit, Santa Maria delle Grazie Hospital, ASL Napoli 2 Nord, 80078 Pozzuoli, Italy; 5 Cardiology Unit, San Giuliano Hospital, 80014 Naples, Italy; 6 Department of Radiology, Istituto Auxologico Italiano IRCCS, San Luca Hospital, University Milano Bicocca, 20149 Milan, Italy

**Keywords:** dual-energy CT, cardiac, applications, review

## Abstract

Dual-energy computed tomography (DECT) represents an emerging imaging technique which consists of the acquisition of two separate datasets utilizing two different X-ray spectra energies. Several cardiac DECT applications have been assessed, such as virtual monoenergetic images, virtual non-contrast reconstructions, and iodine myocardial perfusion maps, which are demonstrated to improve diagnostic accuracy and image quality while reducing both radiation and contrast media administration. This review will summarize the technical basis of DECT and review the principal cardiac applications currently adopted in clinical practice, exploring possible future applications.

## 1. Introduction

Dual-energy computed tomography (DECT) is an emerging imaging technique which consists of the acquisition of two separate datasets utilizing two different X-ray spectra energies (“low kilovoltage peak [kVp]” and “high kVp” spectra).

Any specific material presents different X-ray absorption characteristics at varying levels of energies [1,2]. Outlining these differences, DECT provides many more applications [3] in addition to traditional single-energy CT (SECT), which mostly gains density and anatomical information [4].

Since its first appearance, several cardiac DECT applications have been explored, such as virtual monoenergetic images (VMI), virtual non-contrast (VNC) reconstructions, and iodine myocardial perfusion maps, which are demonstrated to improve diagnostic accuracy and image quality while reducing both radiation and contrast media administration [5,6,7,8].

Although the introduction of DECT dates back to more than a decade ago [9], there has been latency in the widespread adoption of this technology, mainly due to poor availability of scanners, lack of diffuse clinical validation, and shortage of prognostic studies suggesting superiority over conventional imaging techniques.

Currently, several studies are ongoing to further improve the image quality and diagnostic accuracy of DECT, yielding the expansion of cardiac CT applications [10,11,12]. This review will analyze the technical basis of DECT and review the principal cardiac applications currently adopted in clinical practice, exploring possible future applications.

## 2. Basic Principles of DECT

**Figure 1:**
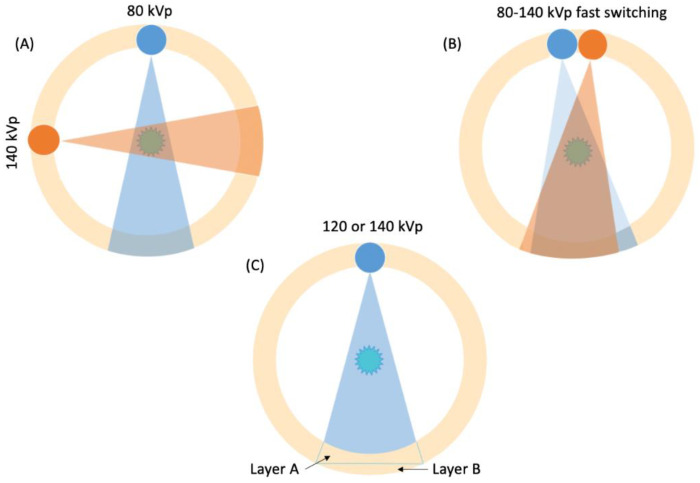
Illustrations showing the main DECT scanning techniques in clinical use. (**A**) Dual-source DECT, consisting of two-source X-ray tubes and the corresponding detectors. (**B**) Single-source DECT consisting of a single tube with rapid kVp switching. (**C**) Detector-based DECT consisting of a single source and a dual-layer detector to obtain low- and high-energy spectra.

Cardiac imaging has always represented a challenging technique due to cardiac and respiratory motion. During the last three decades, technological developments, such as multi-detector row scanners, improved gantry rotation times and acquisition, and post-processing software advancements, unlocked cardiac SECT scans first and have now unlocked DECT [3]. DECT imaging can be obtained through different techniques, which are mainly divided into source-based and detector-based DE [1] (Figure 1).

*Source-based DE* involves the acquisition of CT measurements at two different energy spectra, allowing material characterization via a single or double source. It includes: (a) *dual-spin* mode in which a single tube performs two consecutive sequential scans, and subsequent images overlap; (b) *dual-source (DS)* mode where two orthogonal X-ray tubes working at different kilovoltages manage to perform an accurate spectral separation with subsequent co-registration of the two images datasets obtained; (c) *rapid kVp switching* mode in which a single X-ray tube rapidly varies its kVp; and (d) *twin-beam* mode where a single X-ray spectrum is split into two different energy spectra through a pre-filtration system.

In *detector-based DE*, energy separation occurs at the detector level since the scanner is equipped with a single X-ray source and a multilayer detector. Every layer is manufactured in order to have maximal sensitivity to different photon energies. It mainly includes: (a) *dual-layer CT* where a single X-ray tube is equipped with a dual-layer detector: the top yttrium-based layer absorbs mostly low-energy photons, while the bottom gadolinium oxysulfide layer absorbs the high-energy ones. Thus, through the electronics system, the polyenergetic beam is separated into two energies; (b) *photon-counting CT* uses a cadmium telluride semiconductor detector that is capable of directly converting the X-ray photons into electrical signals. The output signal is proportional to the number of photons, and each photon is allocated to a specific energy bin according to its energy [13].

However, since the heart and coronary arteries are rapidly moving structures, DS-DECT and dual-layer detector DECT are the only two types of DE technology capable of obtaining a complete simultaneous data acquisition with lower risks of misregistration than other strategies [14].

Some of the most critical achievements of DECT were recently obtained through the latest DS generation scanners, which now allow a wider spectral separation between the tubes, with a tube working at 70 kVp and the other at 150 kVp, providing a maximum difference range of 80 kVp.

This improvement means more accurate dual-energy information and improved material differentiation [15]. Furthermore, the introduction of tin-filtration of the X-ray spectrum has allowed an important reduction in dose levels, despite the higher energies applied during acquisition [16]. However, it should be taken into account that low-dose levels reached via SECT for cardiac imaging cannot be obtained by DECT acquisition mode [17,18]. Moreover, DECT technology makes ‘flash’ CT acquisition mode impossible because, as previously reported, DECT needs two X-ray tubes working with different tube voltages while high-pitch mode requires the same voltage on both X-rays tubes [19].

As expected, the amount of raw data for a DECT examination is about twice that of a SECT one. These data allow many different reconstructions that need to be post-processed and interpreted, thus reflecting a very complex and time-consuming workflow for both technologists and radiologists. Hopefully, this workflow is constantly improving thanks to automated or semi-automated post-processing software performed directly at the scanner or workstations, which may significantly reduce the amount of time needed for analysis and interpretation [20].

Finally, the recently introduced photon-counting CT (PCCT) could represent a game-changer in the DECT era. In contrast to the conventional DECT, PCCT has the capability to count both the total number of X-ray photons and their energy distribution, increasing contrast-to-noise ratios and energy-discrimination capabilities. This translates into superior noise characteristics, especially in low-dose scans, that can be very useful in pediatric imaging or screening imaging, such as calcium scoring or the follow-up of dissections. Moreover, the presence of a semiconductor material in place of a scintillator material, and the option to subdivide detector units, can also result in higher spatial resolution. This could be helpful for the evaluation of coronary lumen and stent patency where calcium-blooming and metal artefacts (consequences of partial volume effects of low resolution) can lead to an overestimation of stenosis. Improved spatial resolution can also be useful in better depicting high-risk plaque features, such as thin-cap fibroatheroma or microcalcifications, raising CCT predictive value. PCCT allows an “always available” multi-energy discrimination that can be helpful in significantly reducing metal artefacts and properly image prosthetic valves, stents, ICDs, or any other cardiac devices, consequently improving the assessment of any peri-procedural complications [21].

## 3. Applications

### 3.1. Virtual Monoenergetic Imaging (VMI)

One of the major applications for which DECT represents a turning point in diagnostic imaging is the possibility of processing monochrome reconstructions as if they were obtained using a monoenergetic X-ray beam, hence the name virtual monoenergetic imaging (VMI). This application can improve image quality by optimizing the contrast-to-noise ratio and may help reduce artefacts, the dose of contrast medium, the radiation dose to the patient, and the examination time [22,23,24].

Virtual monoenergetic images are obtained through a post-processing technique that calculates images at the desired level of hypothetical energy (keV). Several studies have shown that getting reconstructions with very low keV, close to the K-edge of iodine (33.2 keV), may improve vessel contrast [6,22]. However, while the effect of reducing keV increases image contrast, it also results in a considerable increase in noise, producing images that cannot be used for diagnosis except at a minimum of 70 keV [25,26].

VMI+ is a new reconstruction algorithm introduced by Grant KL et al. [20]. In detail, the algorithm works through a regional spatial, frequency-based recombination of the high signal at lower energies, and the superior noise properties at medium energies, to escape noise increasing at lower calculated energies. Their method has improved image post-processing, allowing reconstructions up to 40 keV, with a substantial increase in both contrast-to-noise ratio (CNR) and signal-to-noise ratio [20]. Zeng et al. nicely summarized how the application of the VMI+ protocol brings significant benefits in various fields, such as oncology, pulmonary embolisms (Pes), active arterial hemorrhages, and liver lesions [24]. However, several studies reported no effective match between quantitative and qualitative image quality, as few readers prefer images at 50–60 keV due to the excessive increase in noise below this keV, which is not justified by the increase in contrast [22,27].

The absence of a standard processing algorithm and an optimal value of VMI obviously limits the diffusion of this application, especially considering the multitude of factors that influence post-processing operations [6,23,24,28].

Furthermore, De Cecco et al. showed that autonomous window adjustment and reworking results in higher reader confidence and performance, as demonstrated by better liver lesion detection rates in the noise-optimized VMI+ series [29]. Some authors reported differences in reader-preferred and calculated optimized window settings in abdominal DECT angiography [30] and DECT pulmonary angiography [31].

In general, the use of 70 keV VMI reconstructions as the optimal energy level for thoracoabdominal CT angiography images is preferred, while additional reconstructions at 40 or 50 keV may be helpful when poor vascular contrast is noted, as suggested by Albrecht et al. [6].

**Figure 2:**
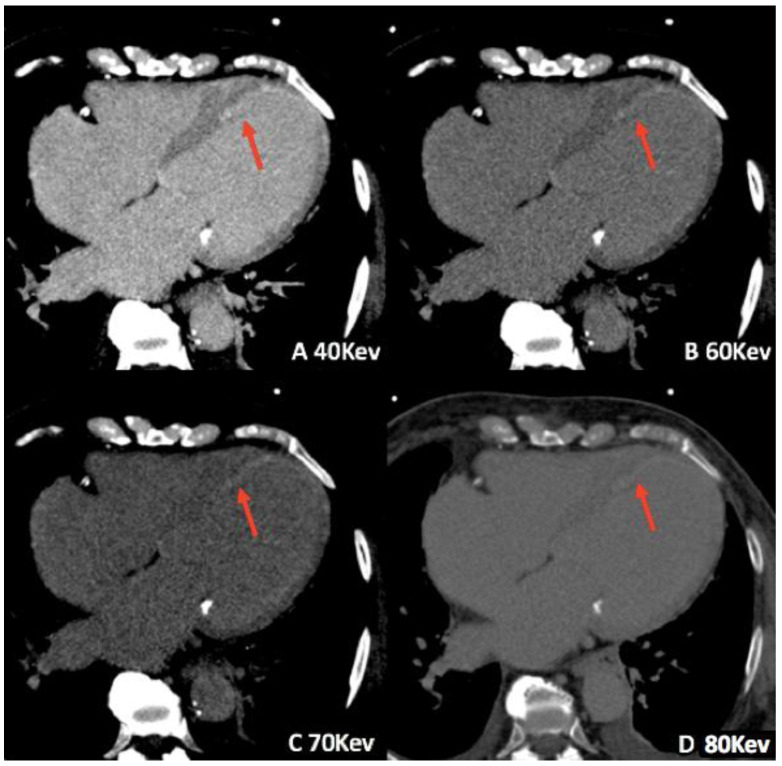
Late iodine enhancement dual-source DECT images in a 72-year-old man. Sub-endocardial hyperenhancement (red arrows) can be seen in the left ventricular septal wall and apex at different virtual monochromatic image values (**A**–**D**).

Nowadays, no large-scale work demonstrates a greater diagnostic accuracy and efficacy of using VMI in cardiac or vascular imaging. However, several papers have highlighted the potential of this application in the field, particularly for the evaluation of late iodine enhancement in cardiomyopathies and myocardial ischemia [32,33] (Figure 2), as well as for detecting active abdominal hemorrhages [34] and endoleaks [35]. Furthermore, recent investigations explored VMI potentials for coronary artery calcium (CAC) scoring [36] and quantifying epicardial adipose tissue attenuation [37], as well as for developing protocols aimed to obtain better quality images with lower radiation doses, especially for transcatheter aortic valve replacement planning [38,39].

**Figure 3:**
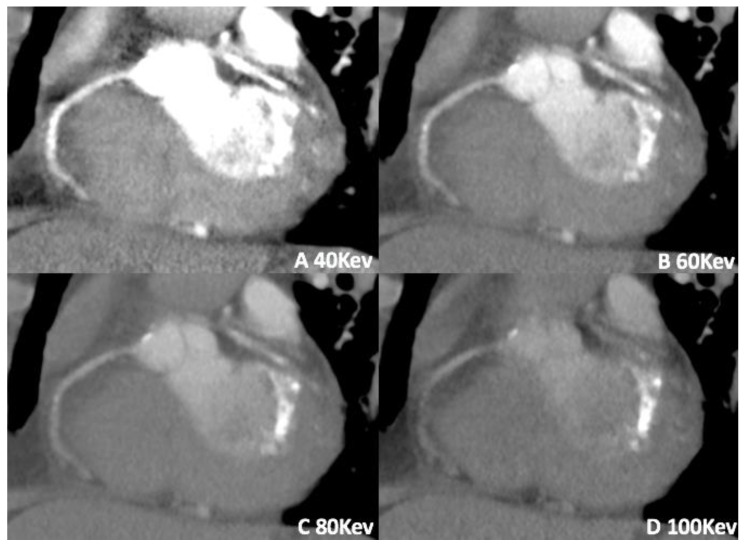
Retrospective application of VMI (**A**–**D**) by dual-source DECT in a patient where the contrast bolus was mistimed in amount; low keV reconstruction increases the attenuation of iodinated contrast material, allowing for greater contrast-to-noise ratio.

Moreover, low keV VMI reconstructions may increase the baseline attenuation of iodine, reducing the amount of contrast medium necessary for the patient [34], as previously demonstrated for vascular abdominal and thoracic CT applications [23,40]. This feature appears to be very helpful when the administration of intravenous contrast medium is troubling, such as in case of heart or renal failure, or for patients with difficult venous access [23] (Figure 3).

Recently, Oda et al. [41] demonstrated that 50 keV VMI enabled a reduction in the contrast medium dose by 50% without coronary CT angiography (CCTA) image quality degradation in patients with renal insufficiency.

Mangold et al. also explored the impact of VMI reconstruction on the evaluation of coronary stents, demonstrating that they may reduce the radiation dose to 49% lower than that of 120 kV SECT [42].

Rotzinger et al. demonstrated that low-keV VMI improves vessel area segmentation in vitro [43]. The same group observed that, in vivo, low keV VMI allows for a 40% iodine dose and injection rate reduction, while maintaining diagnostic image quality, and improves the CNR between lumen versus fat and muscle.

Moreover, Huang et al. [44] showed that 50 keV VMI, compared to polychromatic images, may offer equivalent or improved coronary image quality in CCTA performed on dual-layer spectral detector computed tomography with half the amount of contrast media.

Furthermore, the expertise and subjectivity of the reader determined further elaboration in the adjustment of the window width and level, which is an essential factor, especially in cardiac and coronary imaging, where higher contrast quality is often required [30].

Artefact reduction represents another critical application of DECT. The primary artefacts that affect and reduce the quality of a CCTA examination are beam-hardening and calcium-blooming artefacts. VMI reconstructions can limit both, although in different ways [22].

In myocardial DECT, beam-hardening artefacts predominantly affect the anterior wall of the left ventricle and the descending aorta. Low-energy VMI reconstructions can solve this problem in most cases since the beam cannot harden at higher energy levels [45,46].

Calcium-blooming artefacts often result in non-diagnostic examinations in patients with significant and disseminated coronary calcifications or in an overestimation of the degree of stenosis. The same issue applies to patients with previous coronary stent implantations. SECT image datasets are hampered by metallic devices, such as stents, ICDs, or prostheses, generating beam-hardening and photon-starvation artifacts [47].

High keV VMI obtained through DECT scanners can reduce these artefacts by simply adjusting the monoenergetic level to the optimal value during the post-processing phase. Recent studies demonstrated that the optimal monoenergetic levels to reduce artefacts in a patient with metallic implants range from 105 to 120 keV. In these cases, high keV VMI reconstructions (110 keV and above) can limit beam-hardening artefacts, improving image quality and diagnosis [48,49].

Moreover, since increasing keV results in reduced contrast and overall image quality, it appears useful to obtain reconstructions at different keV levels and optimize the study windows. Accordingly, Ohta et al. [50] showed that maximal contrast-to-noise ratios were observed at 70 keV for calcified and non-calcified plaque and fat in comparison with lumen, while 70 keV and 120 keV were the best VMI keV values for non-calcified plaque in comparison with fat.

### 3.2. Virtual Non-Contrast Imaging

**Figure 4:**
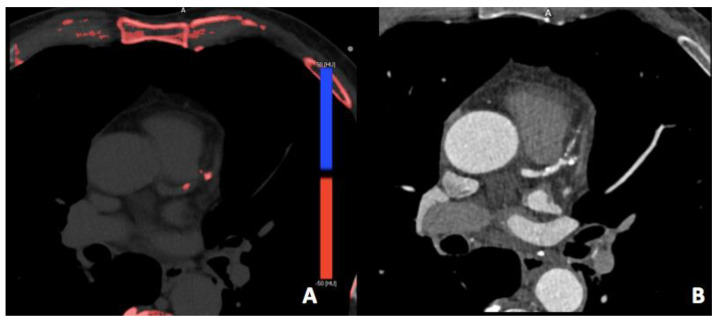
Calcium scoring from coronary dual-source DECT angiography. Virtual non-contrast (**A**) and the corresponding DECT (**B**) images in a 56-year-old man with a virtual Agatston score of 73 on LAD.

DECT can provide selective information on the material and is therefore able to highlight the iodine signal as occurs in perfusion maps, or it can subtract intake, such as for virtual reconstructions without contrast (VNC) [51]. Radiologists can obtain data of the pre-contrast and arterial phases with a single acquisition, thus exposing the patient to a lower dose of radiation without a real non-contrast scan. VNC reconstructions are helpful in differentiating contrast material from calcifications, as well as cardiac and mediastinal structures [23]. The use of high-contrast VNC CT images with dual-energy material decomposition/suppression is also feasible for coronary calcium scoring (Figure 4). Of note, the absolute value of VNC is generally lower than that of true non-contrast-enhanced images [52]. Moreover, the VNC and contrast-enhanced CCTA data are acquired from a single image with a perfect overlay [14], without the possibility of incorrect recordings due to movement artefacts. This allows for an accurate dynamic evaluation of enhancement [23].

### 3.3. Virtual Calcium Subtraction

Material decomposition represents an interesting DECT tool that considers iodine, soft tissues, and calcium as reference materials for coronary arteries analysis. In detail, through the material decomposition algorithm, specific components can be highlighted or subtracted. For coronary artery disease, two pairs of materials are generally used: iodine-calcium and calcium-iodine. The first one allows calcium extraction so that stenosis can be accurately evaluated; in the latter, calcium is removed from the vessel wall while iodine is maintained, yielding a more accurate quantification of the stenosis in the presence of calcified plaques [13,53]. This algorithm is generally integrated with a monochromatic evaluation of the coronaries at high-energy levels (≥80 keV) to reduce blooming and beam-hardening artefacts and offer a precise assessment [54,55]. Recent studies also explored the use of novel calcium-removal image-reconstruction algorithms using PCCT and observed that it had the potential to decrease blooming artefacts caused by heavily calcified plaques, improving lumen evaluation [56,57].

Virtual calcium subtraction reconstructions may improve coronary lumen visualization and diagnostic confidence in patients with heavy coronary calcifications [58]. However, further studies are needed to assess whether this technology could effectively increase diagnostic accuracy.

### 3.4. Iodine Perfusion Maps

**Figure 5:**
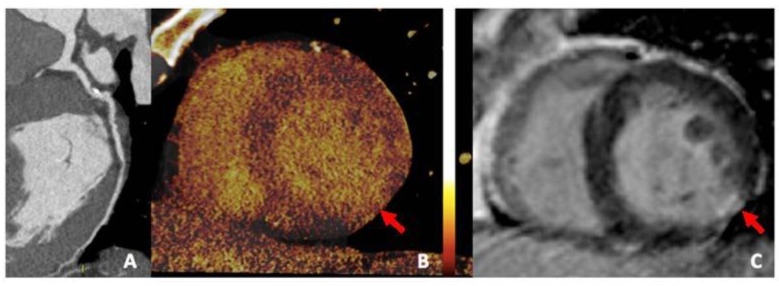
Coronary dual-source DECT: (**A**) Left circumflex coronary artery shows severe proximal stenosis due to the presence of a calcified plaque. (**B**) Iodine perfusion map depicts the perfusion defect of the inferolateral wall (arrow) corresponding to a myocardial scar (arrow) in the short axis LGE MR sequence (**C**), suggestive of necrosis.

Iodine perfusion maps represent one of the most exciting applications of DECT. These maps result from merging iodine-selective reconstruction over the typical anatomical image, and subsequently emphasize the iodine signal manually on the workstation (Figure 5) [1,14,59]. This is based on the principle that gadolinium and iodinated contrast agents share similar kinetics and both can access what had been the intracellular space through the ruptured cell membrane when myocardial necrosis occurs [60].

It is important to underline that DECT perfusion evaluates myocardial blood volume at a single time point instead of using dynamic CT perfusion techniques that allow a multiphase dynamic quantification of myocardial blood flow through time [8]. DECT perfusion generally consists of a static acquisition to visualize the “first-pass perfusion”. Dynamic DECT perfusion is rarely an option for the higher radiation dose due to the multiple acquisitions and because SECT offers a more excellent temporal resolution.

Stress CT is another essential technique for perfusion imaging, even if the best pharmacological strategy is still debated [46]. However, stress image acquisition, before rest to avoid contrast contamination in the myocardium, seems to be the first choice for most centers.

Many recent studies have demonstrated that introducing iodine perfusion maps increases the diagnostic accuracy of cardiac CT scans when compared with cardiac MRI, single-photon computed tomography (SPECT), and invasive catheterization angiography [61,62,63,64,65,66]. By ensuring the quantification of intramyocardial iodine uptake and a better qualitative assessment of myocardial extracellular space, the iodine perfusion maps allow distinguishing ischemia from infarction [67], thus providing functional and anatomical information at the same time.

Myocardial blood volume can be obtained through iodine maps of any cardiac DECT [66]. However, further investigations are needed to establish the optimal acquisition protocol for myocardial infarction and ischemia.

Outlining the iodine distribution in the myocardium, iodine maps favor the radiologist with a better qualitative assessment of perfusion defects, increasing the infarct detection rate, especially when compared with stress CCTA [67,68]. Furthermore, a quantitative assessment of myocardial blood supply can be obtained through an evaluation of myocardial iodine uptake in mg/mL [1,69], with the mean value of iodine concentration different in the ischemic myocardium compared to the healthy or infarcted myocardium [70].

Hence, quantitative and qualitative evaluation through iodine perfusion maps may potentially improve diagnostic accuracy for myocardial infarction and stress-induced ischemia [8,68,69].

Moreover, recent studies demonstrated that iodine perfusion maps could be useful for predicting the hemodynamic significance of coronary artery stenosis, eventually outlining a decrease in iodine uptake in the corresponding territory [67,71]. Furthermore, perfusion maps could play a role in detecting late-enhancing tissue [72] and ultimately favoring differentiation between chronic or reversible myocardial ischemia [14,73]. However, iodine perfusion maps obtained via first-generation DECT scanners typically expose the patients to high radiation; thus, the availability of advanced CT technology is mandatory in this context.

### 3.5. Plaque Imaging and Analysis

Plaque rupture and acute thrombotic coronary occlusion generally occur on thin-cap fibroatheromas, which have been the main target of vulnerable plaque imaging in recent years [74]. As known, CCTA can identify many characteristics associated with vulnerable plaques, such as low attenuation, positive remodeling, spotty calcifications, and the napkin-ring sign [75,76,77].

**Figure 6:**
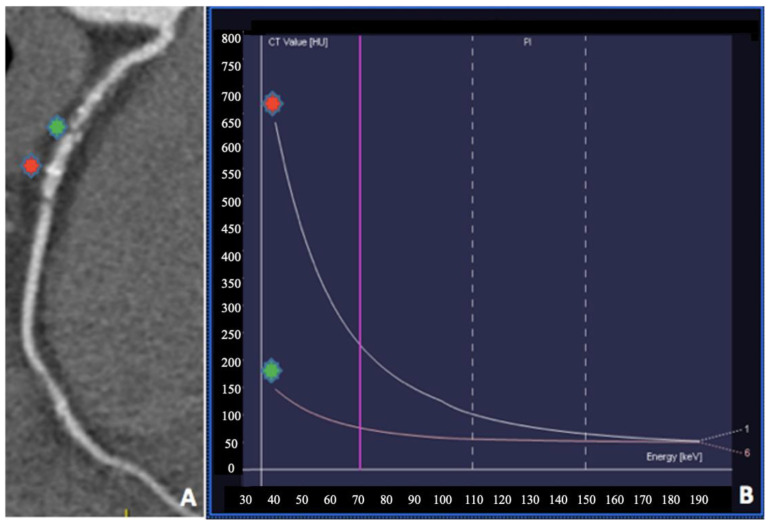
Coronary dual-source DECT in a 65-year-old man presenting with chest pain. (**A**) Automatically generated curved multiplanar reformation of the right coronary artery demonstrates greater than 75% stenosis (green asterisk). (**B**) Plaque analysis using monochromatic coronary reconstruction showing a clear separation of quantitative values (HU) at low keV (red asterisk: calcific plaque; green asterisk: fibrous plaque).

DECT could play a role in evaluating high-risk plaques thanks to its capability to use X-rays at different energies, which impact the attenuation values of different plaque elements, such as fibrous tissue and necrotic core. However, evidence on plaque imaging of DECT is few and often conflicting [23]. DECT may well distinguish calcified- from non-calcified plaques, with no real advantage in the classification of other plaque types compared to the conventional CT [78] (Figure 6). However, in a recent study, Obaid et al. showed that using CCTA at two different energy levels (100 and 140 kV) can improve the sensitivity and specificity for identifying plaque’s necrotic core ex vivo, while the diagnostic accuracy in vivo for the detection of necrotic core is still suboptimal [79].

Furthermore, Tanami et al. demonstrated that the diagnostic performance of CT analysis for ex vivo plaque characterization was superior at lower energy settings (80 kV) to differentiate lipid-rich plaques from fibrotic plaques [80]. Moreover, the ratio of the CT attenuation value at the 80 kV setting divided by the 140 kV setting (Hounsfield ratio [HR], 80:140) could be a practical tool for plaque classification [80].

However, further studies are warranted to explore the potential role of DECT in unravelling the characterization of non-calcified plaques [81]. In the near future, thanks to its higher spatial resolution, PCCT could play a pivotal role in helping to outline high-risk plaques features, such as thin-cap fibroatheroma or microcalcifications.

### 3.6. Extracellular Volume (ECV)

The extracellular volume (ECV) represents a marker of reactive interstitial fibrosis or scar-replacement fibrosis and may represent an added value in several conditions, including both ischemic and non-ischemic manifestations [82]. Although cardiac MRI is the primary imaging modality in this regard, with T1-mapping increasingly adopted in clinical practice, DECT is gaining importance as an alternative technique with good comparability and accuracy [83]. Of note, measurements by DECT are only performed on the iodine maps, thus allowing accurate ECV quantification with minimal erroneous recording [84].

In a recent study, Wang et al. evaluated the feasibility and accuracy of DECT technique in determining the ECV in 35 patients with heart failure, using 3T MRI as the reference standard, showing a good correlation between the two techniques [85].

Ohta et al. evaluated myocardial ECV for each cardiac segment using iodine density image with single-source DECT in 23 patients, using MRI T1 mapping as the reference standard, demonstrating a strong correlation between both methods for either regional or segmental evaluations [86].

## 4. DECT Limitations

As described in previous sections, cardiac DECT presents several advantages, offering new essential applications that can improve the radiologist’s contribution to the clinical setting. However, some disadvantages should be acknowledged. First of all, DECT scanners are expensive, approximately 25% more than an equivalent SECT, as a consequence of the complex hardware manufactured, which raises their cost. Moreover, these scanners need specific complex software to fully exploit the technology, with consequent price elevation. In addition, radiologists and technicians need extensive training to fully take advantage of DECT’s potential.

Furthermore, some limitations are directly linked to the subtypes of multi-energy CT scanners. Due to its lower temporal resolution, rapid kVp switching DECT is prone to motion artefacts, even if this scanner allows excellent energy separation and projection-based VMI reconstructions.

Finally, DS, sequential DECT and twin-beam scanners generally provide higher temporal resolution but with a higher rate of misregistration of the two energy datasets. Instead, dual-layer detector CT permits perfect simultaneous registration of both energy datasets even if it is more prone to misregistration of photons by one of the two layers [87].

## 5. Conclusions

The implementation of DECT in cardiac imaging allows several advantages, mainly consisting of improved image contrast, generation of virtual monoenergetic images, metal artefacts reduction, and virtual calcium subtraction. Moreover, plaque characterization, iodine perfusion maps, and the opportunity to assess late iodine enhancement as well as to quantify ECV complete DECT arsenal. Therefore, DECT represents a cutting-edge technology that is radically changing this field of imaging, going beyond the traditional concepts of material density. Moreover, through its higher spatial resolution and CNR, the recent introduction of PCCT could represent a pathbreaking element for DECT era, allowing novel clinical applications, such as calcium and stent subtraction, which could enhance coronary and in-stent lumen visibility. Further research and technological advantages are expected in the next future to strengthen DECT applications in clinical practice.

## Data Availability

Not applicable.
